# Exploring the role of singing, semantics, and amusia screening in speech-in-noise perception in musicians and non-musicians

**DOI:** 10.1007/s10339-023-01165-x

**Published:** 2023-10-18

**Authors:** Ariadne Loutrari, Aseel Alqadi, Cunmei Jiang, Fang Liu

**Affiliations:** 1 School of Psychology and Clinical Language Sciences, University of Reading, Earley Gate, Reading, RG6 6AL UK; 2 Division of Psychology and Language Sciences, University College London, London, WC1N 1PF UK; 3 Music College, Shanghai Normal University, Shanghai, 200234 China

**Keywords:** Sentence repetition, Music training, Speech perception in noise, Singing, Amusia, Semantics

## Abstract

Sentence repetition has been the focus of extensive psycholinguistic research. The notion that music training can bolster speech perception in adverse auditory conditions has been met with mixed results. In this work, we sought to gauge the effect of babble noise on immediate repetition of spoken and sung phrases of varying semantic content (expository, narrative, and anomalous), initially in 100 English-speaking monolinguals with and without music training. The two cohorts also completed some non-musical cognitive tests and the Montreal Battery of Evaluation of Amusia (MBEA). When disregarding MBEA results, musicians were found to significantly outperform non-musicians in terms of overall repetition accuracy. Sung targets were recalled significantly better than spoken ones across groups in the presence of babble noise. Sung expository targets were recalled better than spoken expository ones, and semantically anomalous content was recalled more poorly in noise. Rerunning the analysis after eliminating thirteen participants who were diagnosed with amusia showed no significant group differences. This suggests that the notion of enhanced speech perception—in noise or otherwise—in musicians needs to be evaluated with caution. Musicianship aside, this study showed for the first time that sung targets presented in babble noise seem to be recalled better than spoken ones. We discuss the present design and the methodological approach of screening for amusia as factors which may partially account for some of the mixed results in the field.

## Introduction

There has been a wide body of research investigating the ability to process and repeat back spoken utterances. The sentence repetition task—a task involving verbatim sentence repetition—can provide direct insights into various facets of linguistic knowledge (Klem et al. 2015; Komeili and Marshall 2013; Polišenská et al. 2015; Riches 2012) and has been extensively used for clinical purposes (Pham and Ebert 2020; Polišenská et al. 2015; Riches 2012). It has been suggested that the task is underpinned by both short- and long-term memory (Riches 2012), but some controversy remains as to how critical the role of memory may be (Klem et al. 2015). It is of note that sentence repetition under optimal laboratory conditions does not reflect the challenges associated with real-life speech processing. A reason for this is that, in a natural auditory setting, listeners typically need to set apart different sound streams and suppress distracting information (Bregman 1994). One such example is following a speaker in a multi-talker environment (Bronkhorst 2000), a common scenario known in the literature as the ‘cocktail party problem’ (Cherry 1953, p. 976).

### Music training and speech processing

Considerable interest has arisen as to whether musically trained listeners are better equipped to process acoustic signals other than music. More specifically, it has been postulated that individuals with music training gain skills that can enhance speech processing (Krishnan and Gandour 2009; Schön et al. 2004; White-Schwoch et al. 2013; Wong et al. 2007). Apart from honing the perception of lexical tones and prosody (Bidelman et al. 2011; Lima and Castro 2011; Moreno et al. 2009; Zhu et al. 2021), music training has been also associated with better phonological processing (Bhide et al. 2013; Habib et al. 2016, but see Bolduc, Gosselin, Chevrette and Peretz 2020) and categorical speech perception (Bidelman 2017; Bidelman and Alain 2015). However, current evidence paints a mixed picture as to whether musicians outperform musically untrained controls when asked to recall spoken and sung material. There is some evidence that musicianship bolsters recall of spoken (Kilgour et al. 2000; Taylor and Dewhurst 2017) or both spoken and sung material (Kilgour et al. 2000). Further, it has been demonstrated that sung material can be better recalled in musically trained patients with Alzheimer’s disease relative to untrained patients (Baird et al. 2017). Nevertheless, it has been also shown that music training fails to afford participants with a compelling advantage (Racette and Peretz 2007), and, although musicians may be better at remembering instrumental music, this may not translate into verbal material (Wilbiks and Hutchins 2020).

### Music training and speech-in-noise perception

If, as some of the evidence above suggests, musicians have indeed enhanced auditory processing and memory, can they better navigate the auditory scenery under adverse conditions? More pertinently, can they outperform musically naïve individuals when processing speech in noise? The answers to these questions also remain inconclusive (Coffey et al. 2017). Back in 2009, a study reported that musically trained individuals performed better on the Hearing in Noise Test (HINT; Nilsson et al. 1994) and the Quick Speech in Noise Test (QuickSIN; Killion et al. 2004), cautiously attributing musicians’ better performance to their enhanced auditory perception, working memory and stream segregation (Parbery-Clark et al. 2009a, b). A similar finding was reported in a longitudinal study also using HINT (Slater et al. 2015) and another study using a different design (Meha-Bettison et al. 2018). However, many other studies have failed to replicate a musicianship advantage for speech perception in noise (Boebinger et al. 2015; Hsieh et al. 2022; Madsen et al. 2017, 2019; Ruggles et al. 2014; Yeend et al. 2017).

Various hypotheses have been put forward in light of these contradictory findings. The musicianship advantage has been partly attributed to more ecologically valid scenarios created through manipulating the location and the masking levels of targets and distractors (Clayton et al. 2016; Swaminathan et al. 2015). However, research with a considerably larger sample size has failed to replicate such findings (Madsen et al. 2019). Music training advantages have been also traced to better processing of momentary fundamental frequency changes during speech segregation (Başkent and Gaudrain 2016), but, again, this has been called into question elsewhere (Madsen et al. 2017). In a different vein, non-verbal intelligence—rather than musicianship itself—was shown to explain better speech-in-noise perception in a study (Boebinger et al. 2015), but such an account has been refuted by both prior and subsequent work (Parbery-Clark et al. 2009a, b; Slater and Kraus 2016). As outlined below, unresolved questions surrounding the purported musicianship advantage have not been considered in relation to the target’s semantic content and its mode of presentation.

### The role of semantics in recall and repetition

With different target sentences being used across studies, variation in semantic content (and/or discourse) could partially account for some of the discrepancies observed. Evidence suggests that readers tend to better recall information in a narrative relative to an expository form (Kintsch and Young 1984; Zabrucky and Moore 1999), perhaps owing to reminiscences of everyday life events (Gardner 2004). A recent meta-analysis has backed up these findings while noting that content complexity across semantic categories has not been appropriately controlled for in the original studies (Mar et al. 2021). In the oral domain, immediate recall of semantically plausible sentences appears to be enhanced relative to semantically implausible ones (Polišenská et al. 2015, 2021), and even when it comes to multiple concurrent auditory sources, processing demands can be moderated by semantic expectancies (Golestani et al. 2010; Obleser and Kotz 2010). The semantic content of target sentences used in many studies looking at speech-in-noise perception in musicians and non-musicians has been generally kept simple as in, ‘She cut with her knife’ or ‘The sense of smell is better than that of touch’ (Başkent and Gaudrain 2016; Boebinger et al. 2015; Parbery-Clark et al. 2009a, b; Slater and Kraus 2016). Other studies in this area have used short sentences of not particularly meaningful—albeit not anomalous—semantic content (Clayton et al. 2016; Swaminathan et al. 2015). It is not, however, known whether controlling for semantic variation in a single study can have a direct bearing on speech-in-noise perception findings.

### Singing as a previously unexplored variable

A question that has been overlooked in the field is whether a sung target can lead to differences in sentence repetition. Despite both spoken and sung stimuli involving vocal information, their processing entails not only different vocal control (Natke et al. 2003) but also dissimilar temporal organization (Kilgour et al. 2000; Lehmann and Seufert 2018). As opposed to the speech modality, singing affords listeners with additional salient cues, such as melody and rhythm (Sloboda 1985). Further, due to its typically slower time window (Patel 2014), sung material can be recalled more efficiently (Kilgour et al. 2000), but there is also evidence to the contrary (Racette and Peretz 2007). Musicians have been shown to better segregate simultaneous sounds (Zendel and Alain 2009) and interleaved melodies (Marozeau et al. 2010), which could place them in an advantageous position for processing singing in background noise. Although such prediction is not universally upheld by the data, it is tenable that the presence of salient cues and slower time organization can lead to better word encoding and, in turn, more accurate repetition in quiet and in noise, with such effect being more pronounced in musicians.

### Amusia screening as a new methodological approach

By a similar token, the question arises as to whether music deficits can affect speech perception and group comparisons based thereon. Comparing musicians to control groups that may contain participants with congenital amusia could inadvertently amplify group differences. Typically associated with impairments in pitch perception and production (e.g., Ayotte et al. 2002; Dalla Bella et al. 2009; Foxton et al. 2004; Loutrari et al. 2022), congenital amusia has been shown to occur alongside speech perception impairments detected under laboratory conditions (e.g., Li et al. 2019; Liu et al. 2015; Sun et al. 2017; Zhang et al. 2017). However, previous work on the role of musicianship in speech perception in noise has not considered potentially confounding amusia-related effects, which may lead to unwarranted variability in control samples. Screening for amusia can therefore ensure more reliable group comparisons.

### The present study

In this study, we adapted the Sentence Repetition Task by introducing trials with babble noise, including three different semantic categories, and incorporating spoken and sung targets. In the light of previous research showing that semantically anomalous speech poses additional difficulties and recall of written language is differentiated by genre, our version of the task involved three types of sentences: news-like sentences (hereafter ‘expository’), sentences from stories (‘narrative’) and sentences violating semantic rules (‘anomalous’). We predicted that semantically anomalous sentences would be less efficiently recalled and that performance would be higher for narrative content. Regarding spoken versus sung sentences, we hypothesized that, as speech and music were presented in an ecologically valid form—without equalizing the duration of stimuli across conditions—words of sung sentences would be perceived better than spoken ones both in quiet and in noise. Given the mixed findings in the literature, we did not have a specific prediction as to whether musicians would outperform non-musicians on the Sentence Repetition Task. We nevertheless sought to address whether possible cases of amusia would have an effect on group comparisons given prior evidence associating amusia with speech comprehension difficulties in quiet and noisy conditions (Liu et al. 2015). To this end, we screened participants for music perception impairments, administering the Montreal Battery of Evaluation of Amusia (MBEA) (Peretz et al. 2003), while also looking at performance on non-musical cognitive tests. While the MBEA is typically used to detect perceptual music deficits, some of its subtests have been previously used in studies with musicians and non-musicians (Habibi et al. 2014; McAuley et al. 2011; Scheurich et al. 2018).

## Methods

### Participants

One hundred participants took part in the main portion of the study, of which 27 completed the experiment in the laboratory. The rest of the participants (*n* = 73) were recruited on prolific.co, an online recruitment platform. Participants were remunerated for two hours of their time. They were all monolingual native British English speakers with normal hearing (confirmed by hearing screening in the laboratory, or self-reported online), aged between 18 and 46 years.

Those who had at least six years of formal music training (*n* = 50) were classified as musicians, whereas non-musicians (*n* = 50) had two years or less of music training, in line with the musicianship criteria applied in other studies (e.g., Madsen et al. 2017; Weijkamp and Sadakata 2017; Xie and Myers 2015). Years of training were added up if a participant played more than one instruments (Pfordresher and Halpern 2013); for instance, five years learning the violin and three years learning the piano were recorded as eight years of music training. A considerable training difference was seen between groups, as musicians had a long period of music training, whereas most non-musicians had received no music training.

All participants were screened for amusia using the MBEA (see the Materials section for more details on the battery). Those with a pitch composite of 65 or less (Liu et al. 2010) and/or a global score of 71% or less (Nan et al. 2010) were classified as amusics. The screening led to the detection of thirteen amusic cases. Surprisingly, two of these 13 participants were in the musically trained group.

As a large number of amusic participants was detected, we sought to explore whether amusic individuals would perform significantly worse as a group. To this end, we recruited additional amusic participants, this time explicitly calling for people with music difficulties. Combining data from these two separate recruitment rounds led to a total of 27 amusic participants. Their data were inputted in a separate model comparing amusic participants to an equal number of matched non-musician controls (see the Results subsection on sentence repetition in amusic participants). All but three of these participants were recruited online; only three in-laboratory control participants were included to ensure that the groups, namely amusics and controls, were matched for gender and age. It should be noted that the two amusic musicians mentioned earlier were not included in the amusic group, as amusic participants were compared to non-musician controls; they were only included in the musician group in the main analysis (whereby amusia criteria were not considered). Participant demographics are presented in Table 1.

**Table 1:** Participant demographics

	Group 1	Group 2	*p*
	Musicians (*n* = 50)	Non-musicians (*n* = 50)	
Age (range) (mean)	18–4523.24 (4.53)	19–4624.08 (5.11)	0.40
Gender	38 F, 12 M	40 F, 10 M	0.80
Years of music training (mean)	**15.68 (9.65)**	**0.14 (0.49)**	**< 0.001**
	Amusics (*n* = 27)	Non-amusic controls (*n* = 27)	
Age (range) (mean)	21–4329.22 (6.51)	19–4626.40 (7.85)	0.16
Gender	21 F, 6 M	23 F, 4 M	0.72
Years of music training (mean)	0	0.07 (0.37)	0.32

The table presents details on age, gender and music training with SD in parentheses. *T*-tests were used to compare age and music training, and a *χ*^2^ test was used to compare gender distribution between the groups. Significant differences appear in bold

In addition to the main experimental portion of the study, a number of background measures were also obtained. Short-term memory was assessed using the forward digit span task adapted from the Wechsler Adult Intelligence Scale IV (WAIS IV). Online participants completed the Deary–Liewald task (Deary et al. 2011), which is known to correlate with general intelligence (Deary et al. 2001, 2011), and in-laboratory participants were administered Raven’s Standard Progressive Matrices (Raven and Raven 2003). All participants also completed the Montreal Battery of Evaluation of Amusia (MBEA) (Peretz et al. 2003), a standardized battery consisting of six subtests: scale, contour, interval, rhythm, meter and incidental musical memory. Musicians and non-musicians performed similarly on the non-musical cognitive tests, but musicians outperformed their untrained counterparts on all but one MBEA measure. Participants’ performance on background measures is shown in Table 2.

**Table 2:** Performance of musicians and non-musicians on background tasks

Task		Musicians	Non-musicians	*t*	*p*
MBEA	Scale	**26.28 (2.27)**	**25.70 (2.48)**	**1.20**	**0.23**
	Contour	**25.80 (2.39)**	**23.88 (3.47)**	**3.19**	**0.001**
	Interval	**25.50 (3.14)**	**23.60 (2.92)**	**3.10**	**0.002**
	Rhythm	**25.80 (3.30)**	**24.02 (3.77)**	**2.48**	**0.01**
	Meter	**27.44 (4.05)**	**21.10 (6.36)**	**5.88**	** < 0.001**
	Memory	**27.46 (2.93)**	**25.66 (3.97)**	**2.55**	**0.01**
	Pitch composite	**77.58 (6.56)**	**73.18 (7.14)**	**3.17**	**0.001**
	Global MBEA	**0.87 (0.06)**	**0.80 (0.09)**	**5.03**	** < 0.001**
Deary–Liewald					
(Online only)	Simple trials	99.30 (2.09)	98.38 (4.35)	1.14	0.25
(Online only)	Choice trials	94.26 (8.91)	94.66 (5.32)	0.22	0.82
Raven’s Standard Progressive Matrices (laboratory only)		52.38 (4.76)	52.43 (3.16)	0.02	0.98
Digit span		6.2 (1.44)	6.08 (1.44)	0.41	0.68

Means, SDs and *t*-test results for all background measures, uncorrected for multiple comparisons. In-laboratory participants completed Raven’s Standard Progressive Matrices instead of the Deary–Liewald task. All other measures include data from both in-laboratory and online participants. MBEA subtest scores correspond to the number of correct responses out of 30; the pitch composite score is the sum of the scale, contour and interval subtests; Global MBEA refers to the average percentage of correct responses across subtests. Significant differences appear in bold

Amusic participants’ performance on the same background measures is presented in Table 3.

**Table 3:** Performance of amusic participants and matched non-musician controls

Task		Amusics	Controls	*T*	*P*
MBEA	Scale	**21.56 (4.41)**	**26.33 (2.24)**	**5.02**	**< 0.001**
	Contour	**19.22 (3.14)**	**25.11 (2.79)**	**7.28**	**< 0.001**
	Interval	**18.96 (2.52)**	**24.85 (2.32)**	**8.94**	**< 0.001**
	Rhythm	**20.04 (3.82)**	**24.78 (3.63)**	**4.67**	**< 0.001**
	Meter	**15.44 (5.48)**	**23.41 (5.33)**	**5.41**	**< 0.001**
	Memory	**21.44 (5.28)**	**27.15 (2.21)**	**5.17**	**< 0.001**
	Pitch composite	**59.74 (7.47)**	**76.30 (5.36)**	**9.36**	**< 0.001**
	Global MBEA	**0.65 (0.08)**	**0.84 (0.06)**	**9.54**	**< 0.001**
Deary–Liewald	Simple trials	97.22 (5.60)	98.52 (2.33)	1.11	0.27
	Choice trials	95.28 (7.76)	93.96 (5.37)	0.72	0.47
Digit span		5.81 (0.79)	6.07 (1.36)	0.85	0.40

Note: Means, SDs and *t*-test results for all background measures, uncorrected for multiple comparisons. See also Table 1 for additional information on the two groups compared in this table. Significant differences appear in bold

Ethical approval was obtained from the research ethics committees at the University of Reading and Shanghai Normal University. Written informed consent was obtained from all participants included in the study.

### Materials

The online version of the study was designed in Gorilla, a now widely used online behavioral experiment builder originally launched in 2016 (for more details on Gorilla, see Anwyl-Irvine, et al. 2018). Praat (Boersma 2001) and other software programmes were used for the in-laboratory experiment.

*The Sentence Repetition Task*. This task required participants to listen to different sentences and repeat them back. The output of the in-laboratory participants was recorded in Praat and that of online participants was recorded in Gorilla. Participants were prompted to record their responses by pressing a button. The task consisted of 60 experimental trials and six practice trials. Sentences had an approximate average length of ten words (*M* = 9.91, *SD* = 2.32). Participants were encouraged to repeat at least some of the words to the best of their ability when they could not repeat back a whole sentence. Three types of sentences were included: expository, narrative and semantically anomalous (see Supplementary Material for all sentences). Expository sentences were news-like sentences presenting facts. Narrative sentences were taken from stories. Semantically anomalous sentences were meaningless sentences violating typical semantic expectancies. Expository and narrative sentences were both grammatically and semantically correct. Semantically anomalous items were syntactically well-formed sentences with semantically meaningless content. Word frequency was computed using the Zipf frequency scale of the SUBTLEX-UK word frequency database (Van Heuven et al. 2014). No significant differences in word frequency were observed across semantic categories, *F*(2, 593) = 2.33, *p* = 0.10, *η*_p_^2^ = 0.008. The type of presentation varied. Target sentences had a spoken and a sung version, and they were presented either in quiet or with babble noise. Sung sentences had to be repeated back in the speech modality, that is, participants were not required to sing even if the target was sung. Table 4 presents a summary of acoustic features extracted from spoken and sung targets.

**Table 4:** Summary of acoustic features across spoken and sung stimuli before noise manipulation

Target	Mean F_0_ (Hz)	Min F_0_ (Hz)	Max F_0_ (Hz)	Mean duration (sec)	Mean tempo (bpm)
Speech	242.68	150.14	316.82	2.61	NA
Music	301.50	184.18	385.73	5.53	113

A Praat script, ProsodyPro (Xu 2013) was employed to obtain information on fundamental frequency and duration, and MixMeister BPM Analyzer (MixMeister Technology, LLC) was used to determine the tempo of the sung stimuli. Note that minimum and maximum fundamental frequency values refer to the averaged minimum and maximum frequencies across stimuli (rather than individual data points)

Babble noise was obtained from a previously published 56-speaker data set (Valentini-Botinhao et al. 2016). The signal-to-noise ratio for the stimuli presented with babble noise was set at 5 dB using the MixSpeechNoise.praat script (McCloy 2015). All stimuli, regardless of whether they were presented in noise or in quiet, were normalized to have a root-mean-square (RMS) amplitude of 0.1 Pascal (= 74 dB) using a custom-written Praat script.

All spoken and sung sentences were recorded with a sampling rate of 44,100 Hz by a musically trained female native speaker of British English in a sound booth using Praat. The speaker was an amateur singer, and all sung sentences were produced without vibrato. Four lists were created using a Latin square design and no sentence appeared more than once. These different versions were presented to allow for a different order in terms of the presence/absence of background noise and the type of presentation (speech versus song). The stimuli were presented at a comfortable listening level set by the participants through Sennheiser HD 280 Pro Headphones in the laboratory. Online participants used their personal headphones/devices.

Recorded responses were transcribed and scored by five research assistants. All correctly recalled words were given equal credit regardless of whether participants recalled them in the wrong order and/or added extra words. Participants were penalized for derivational errors (e.g., saying ‘explain’ instead of ‘explanation’) but not for inflectional errors (e.g., saying ‘play’ instead of ‘played’). The analysis was performed using the percentage of correctly recalled words transformed into Rationalized Arcsine Units (RAU), an approach taken by previous studies (Liu et al. 2015; Madsen et al. 2017), as it renders the data more suitable for statistical analysis (Studebaker 1985).

*The MBEA* (Peretz et al. 2003). The MBEA requires participants to perform same/different discrimination judgements comparing pairs of melodies that differ in terms of either pitch (scale, contour and interval) or rhythm. In the fifth subtest, the meter test, participants are instructed to judge whether harmonized sequences are marches or waltzes. The final test assesses incidental memory and participants need to indicate whether they have heard a given melody throughout the course of the trials. Both in the laboratory and online, participants were required to click on a button depending on the response format. The scale, contour, interval and rhythm subtests included 31 trials, one of which was a catch trial. The meter and memory subtests included 30 trials each. In addition to the experimental trials, we included two to four practice trials at the beginning of each subtest. The maximum possible score for each subtest was 30. In addition, we computed the sum of the three pitch subtests (composite score) and the average percentage of correct responses across subtests (MBEA Global).

*The Forward Digit Span (Wechsler Adult Intelligence Scale IV).* In the online version of the task, participants were visually presented with a gradually increasing number of digits and were asked to type the digit sequence they were exposed to in the correct order. The digits ranged from two to nine, with two trials per digit length leading to a total of 16 trials. When participants failed to provide the correct sequence twice in a row, no additional trials were included. The in-laboratory version of the task was designed and presented in a similar fashion but was administered using the Psychology Experiment Building Language (PEBL) (Croschere et al. 2012).

*The Deary–Liewald task* (Deary et al. 2011). This task comprised two parts. In the first part, consisting of two practice items and 20 trials, participants were required to press the spacebar on their keyboard as soon as they saw a diagonal cross within a square. The cross disappeared once the spacebar was pressed and another cross subsequently appeared. The second part involved choice trials; participants were presented with four boxes and were requested to press different keys depending on the box in which a diagonal cross appeared. After completing five practice trials, participants went on to complete 40 experimental trials. The sum of correct responses was extracted for each of the two parts.

### Procedure

Online testing lasted approximately 90 to 100 min, but participants had two hours at their disposal and were encouraged to take breaks between tasks. They were initially asked to provide background information, including their age, gender, medical profile, and music background. A detailed task description and a consent form were subsequently presented. Participants were initially administered the Forward Digit Span Task and the Sentence Repetition Task and moved on to the MBEA followed by the Deary–Liewald Task. Finally, participants were debriefed on the experiment and had the opportunity to make comments and report technical difficulties and/or other concerns. In the laboratory, participants completed the background questionnaire, Raven’s Standard Progressive Matrices (Raven and Raven 2003), the Forward Digit Span Task, and the MBEA as part of background measures collected for a larger project, where participants also took part in the Sentence Repetition Task as well as other experiments during different visits.

### Data analysis

Statistical analysis was conducted using R (R Core Team 2019). Linear mixed-effect models were fitted using restricted maximum likelihood (REML) estimation. Model assumptions were assessed by visually inspecting the residuals. Group (musicians versus non-musicians), presentation type (speech versus song), noise condition (quiet versus babble), and semantic content (expository, narrative and semantically anomalous) were modeled as fixed effects. Note that the sentences used in the quiet and the babble conditions were identical. Sentence length (in terms of number of syllables per sentence) was kept similar across presentation type and semantic content conditions. Length was originally included in the model but had no significant effect on results and no interactions with other variables and was subsequently dropped. Participants and sentences were included as random effects. Interactions among group, presentation type and semantic content were also fitted into the model. To conduct the analysis, we used the lme4 (Bates et al. 2015), car (Fox 2008), lmerTest (Kuznetsova et al. 2017), and emmeans (Lenth 2020) packages. When post hoc comparisons were conducted, *p*-values were adjusted using the Holm method. Figures were designed using the ggplot2 package (Wickham 2011).

One participant was identified as an outlier following visual inspection of the data. The participant had not attempted a large number of trials and was therefore excluded from the analysis reporting on 100 participants.

## Results

### Interim analysis: comparing in-laboratory and online data

Prior to full data collection, we compared in-laboratory participants to an equally sized group of online participants to evaluate the reliability of testing sentence repetition online. More specifically, we compared 14 in-laboratory to 14 online non-musicians and 13 in-laboratory to 13 online musically trained participants. The performance of non-musicians was not associated with significantly different repetition accuracies across testing modalities, *t*(26) = 0.63, *p* = 0.53, and the same result was seen in the musically trained groups, *t*(24) = 0.33, *p* = 0.74.

### Sentence repetition task

Online and in-laboratory data were combined using results from a total of 100 participants (although see Procedure and Sentence repetition in amusic participants for some additional testing outside the main scope of the study). The model revealed a significant main effect of group, with musicians performing better relative to non-musicians, *F*(1, 98) = 4.31, *p* = 0.04, *η*_p_^2^ = 0.04. However, following screening for amusia, 13 participants were excluded from the sample. Rerunning the model without the amusic participants showed that musicians’ performance did not differ statistically from non-musicians, *F*(1, 85) = 2.33, *p* = 0.13, *η*_p_^2^ = 0.02. The rest of the analysis was conducted on the full pool of participants (but see the Results subsection on sentence repetition in amusic participants for a different comparison).

Unsurprisingly, results showed a significant main effect of noise, *F*(1, 5817.48) = 2742.52, *p* < 0.001, *η*_p_^2^ = 0.32. The mode of presentation also came out significant, albeit with a small effect size, *F*(1, 5817.76) = 20.50, *p* < 0.001, *η*_p_^2^ = 0.003, with sung targets being recalled more successfully than spoken ones. A significant mode of presentation × noise level interaction was seen, *F*(1, 5821.47) = 52.23, *p* < 0.001, η_p_^2^ = 0.009, and post hoc pairwise comparisons showed that sung targets were better recalled than spoken ones only in noise, *t*(5822) = 7.22, *p* < 0.001, *d* = 0.09.

The mode of presentation also interacted with semantic content, *F*(2, 5818.11) = 5.29, *p* = 0.005, *η*_p_^2^ = 0.001. Although no differences were seen for narrative and anomalous content, sung expository targets were associated with better performance relative to spoken ones, *t*(5820) = 5.55, *p* < 0.001, *d* = 0.07.

Semantic content also interacted with noise level, *F*(2, 5817.44) = 8.88, *p* < 0.001, *η*_p_^2^ = 0.003, with anomalous sentences being recalled significantly worse than expository sentences in the presence of noise, *t*(5767) = 2.70, *p* = 0.01, *d* = 0.03.

Finally, there was a significant interaction between presentation, noise level, and semantic content, *F*(2, 5819.60) = 4.52, *p* = 0.01, *η*_p_^2^ = 0.002. Further analysis generated 66 FDR (false discovery rate) corrected post hoc comparisons in line with the already presented results; these are omitted here for the sake of brevity.

**Fig. 1:**
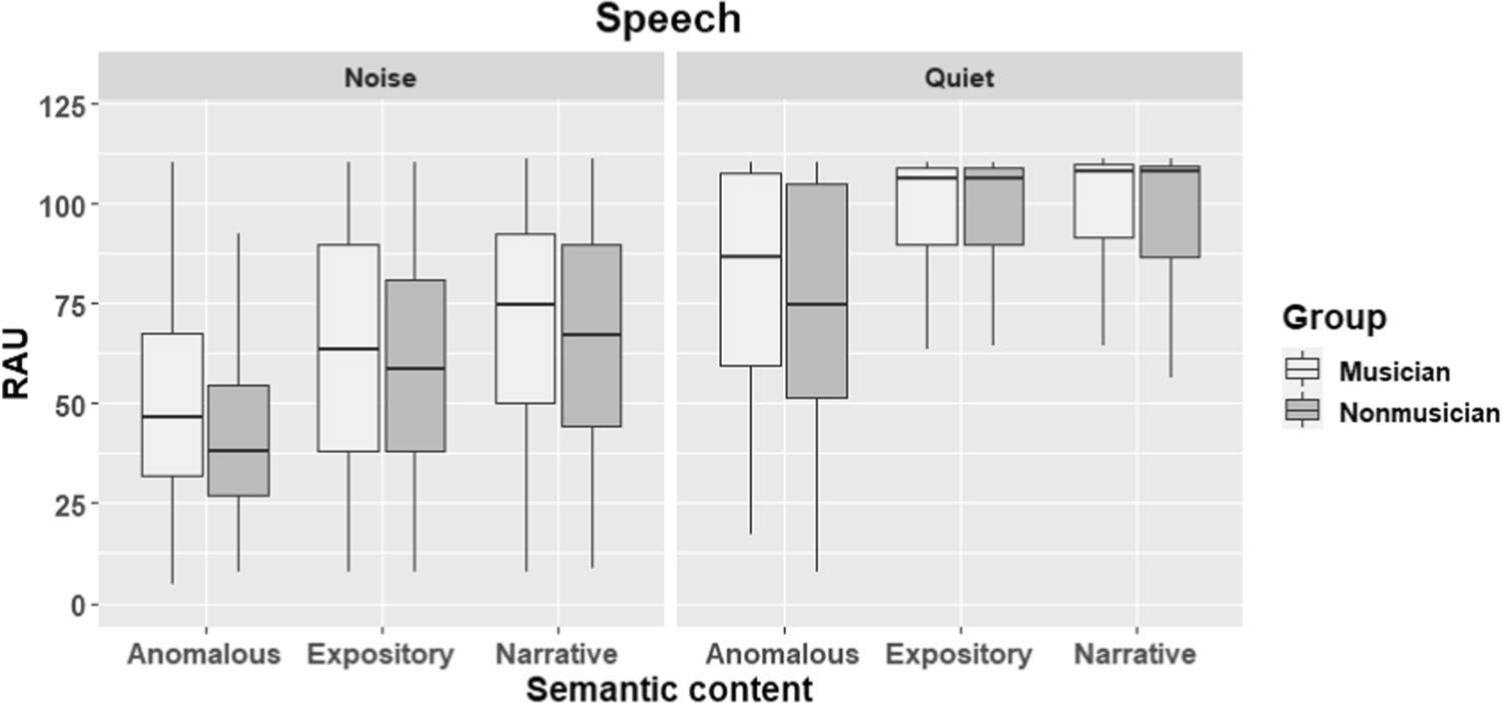
Boxplots showing performance of musicians and non-musicians on all trials of the speech condition. Higher scores reflect higher accuracy. The whisker boxes show the median (thick horizontal line) and the quartiles

**Fig. 2:**
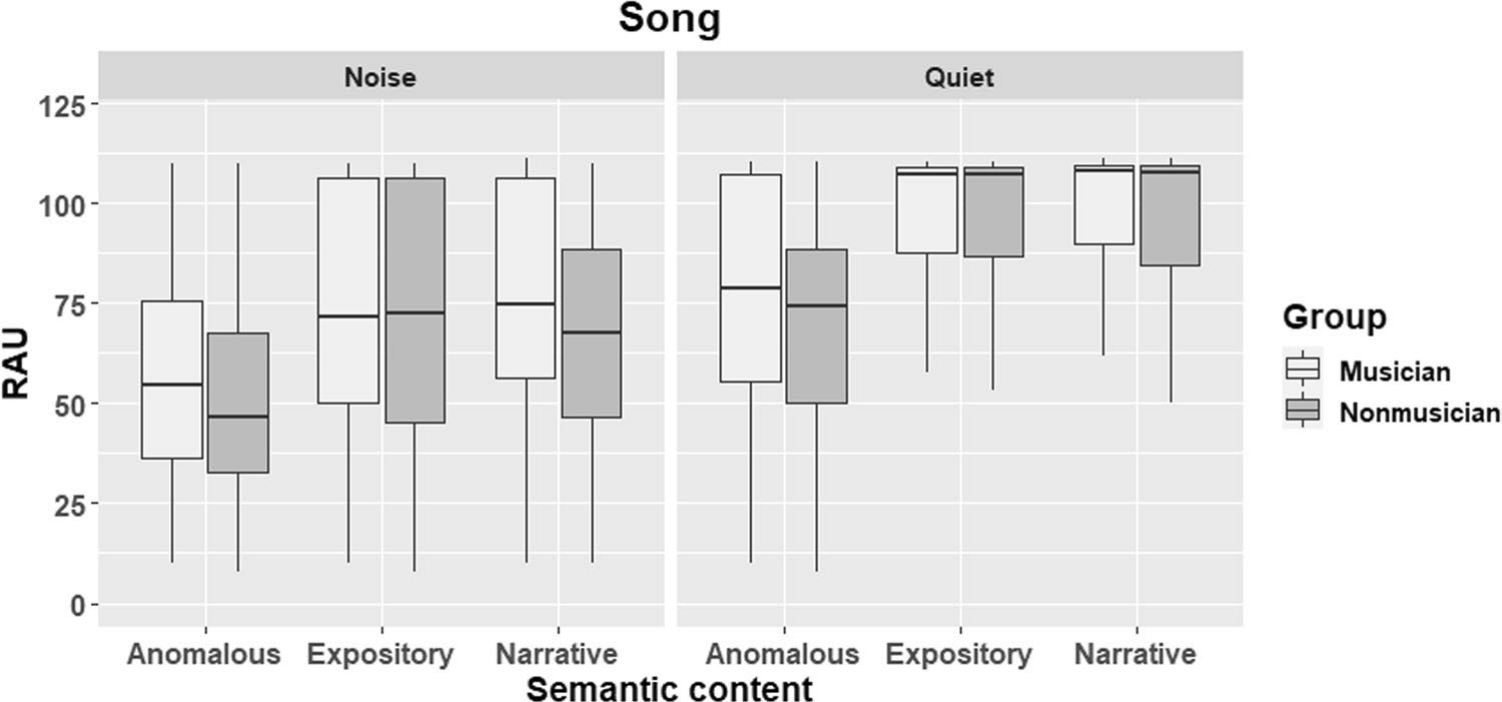
Performance of musicians and non-musicians on all trials of the song condition. The boxplots show the distribution of the data including the median and the quartiles

Results from all trials are visually depicted in Fig. 1 (speech condition) and Fig. 2 (song condition).

The full set of findings, including non-significant results, is shown in more detail in Table 5.

**Table 5:** Results from the linear mixed-effect model including main effects and interactions

	NumDF	DenDF	*F*	*p*	*η*_p_^2^
Group	**1**	**98.00**	**4.31**	**0.04**	**0.04**
Group (following amusia screening)	1	85.00	2.33	0.13	0.02
Presentation	**1**	**5818.77**	**20.50**	**< 0.001**	**0.003**
Noise level	**1**	**5817.48**	**2742.52**	**< 0.001**	**0.32**
Semantic content	2	5440.68	0.25	0.78	< 0.001
Group × Presentation	1	5820.43	0.24	0.62	< 0.001
Group × Noise level	1	5817.48	1.65	0.20	< 0.001
Presentation × Noise level	**1**	**5821.48**	**52.23**	**< 0.001**	**0.009**
Group × Semantic content	2	5823.19	1.19	0.30	< 0.001
Presentation × Semantic content	**2**	**5818.11**	**5.29**	**0.005**	**< 0.001**
Noise Level × Semantic content	**2**	**5817.44**	**8.89**	**< 0.001**	**0.003**
Group × Presentation × Noise level	1	5817.49	0.36	0.55	< 0.001
Group × Presentation × Semantic content	2	5819.28	2.09	0.12	< 0.001
Group × Noise level × Semantic content	2	5817.61	1.99	0.14	< 0.001
Presentation × Noise level × Semantic content	**2**	**5819.60**	**4.52**	**0.01**	**0.002**
Group × Presentation × Noise level × Semantic content	2	5818.11	0.96	0.38	< 0.001

Note: NumDF, numerator degree of freedom; DenDF, denominator degree of freedom; Presentation, mode of presentation. The second group comparison was conducted following the exclusion of amusic participants. Significant effects and interactions are in bold

### Correlations

**Fig. 3:**
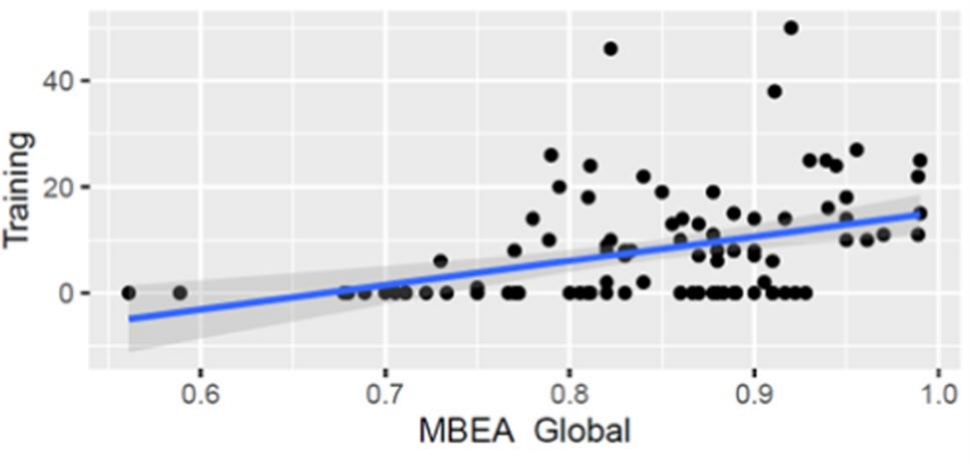
Correlation between years of training and overall performance on the MBEA (*r* = 0.40, *p* < 0.001). Note that training indicates the total years of music training a participant had taken, with more instruments adding up to a larger value

We correlated MBEA Global scores with years of music training (Fig. 3) to gauge whether self-report measures would be reflected in the obtained scores; a significant relationship was indeed found (*r* = 0.40, *p* < 0.001). To explore potential links between music perception and other aspects of cognitive ability, we correlated MBEA Global with digit span and the Deary–Liewald choice trials. No significant correlations were seen for either MBEA Global and digit span (*r* = 0.13, *p* = 0.16) or MBEA Global and the Deary–Liewald task (*r* = 0.11, *p* = 0.33). Similarly, no significant correlations were observed between years of music training and digit span (*r* = 0.08, *p* = 0.44) or years of music training and Deary–Liewald (*r* = 0.07, *p* = 0.54).

### Sentence repetition in amusic participants

As mentioned earlier, to explore whether amusic individuals would perform significantly worse as a group on sentence repetition than non-musician controls, we compared 27 amusics with an equal number of matched non-musician controls. No significant group differences were found when comparing amusic participants to controls, *F*(1, 50.91) = 1.61, *p* = 0.21, *η*_p_^2^ = 0.03. Similar to the first model (comparing musicians to non-musicians), results revealed a significant effect of noise, *F*(1, 3107.99) = 1645.58, *p* < 0.001, *η*_p_^2^ = 0.35, with higher scores for sentences presented in quiet, and mode of presentation, *F*(1, 3102.82) = 5.69, *p* = 0.02, *η*_p_^2^ = 0.002, with better performance for sung sentences.

The second model also corroborated the three significant interactions seen in the first model. Mode of presentation interacted significantly with noise level, *F*(1, 3100.3) = 28.32, *p* < 0.001, *η*_p_^2^ = 0.009. Post hoc pairwise comparisons showed that sung sentences were recalled significantly better than spoken sentences in noise, *t*(3107) = 5.44, *p* < 0.001, *d* = 0.09, whereas the opposite pattern was observed in quiet, albeit with a smaller effect size, *t*(3107) = 2.06, *p* = 0.04, *d* = 0.03.

The mode of presentation interacted significantly with semantic content, *F*(2, 3102.04) = 4.48, *p* = 0.01, *η*_p_^2^ = 0.003, with better performance associated with sung expository relative to spoken expository sentences, *t*(3108) = 3.75, *p* = 0.002, *d* = 0.06, and no significant differences across the other categories.

Semantic content also interacted with noise level, *F*(2, 3106.57) = 15.46, *p* < 0.001, *η*_p_^2^ = 0.01. In quiet, expository sentences were recalled more accurately than anomalous sentences, *t*(2612) = 2.78, *p* = 0.007, *d* = 0.05, whereas this was not seen in noise, *t*(2612) = 1.28, *p* = 0.23, *d* = 0.02. In a similar vein, narrative sentences were recalled significantly better than anomalous sentences in quiet, *t*(2612) = 2.85, *p* = 0.006, *d* = 0.05, but not in noise, *t*(2612) = 1.85, *p* = 0.08, *d* = 0.03.

A significant interaction was observed this time between group and semantic content, *F*(2, 3122.96) = 4.18, *p* = 0.02, *η*_p_^2^ = 0.003, but FDR-corrected pairwise comparisons did not reveal any significant differences.

More details on the comparison between amusic participants and controls are presented in Table 6.

**Table 6:** Results from the linear mixed-effect model comparing amusic participants and controls

	NumDF	DenDF	*F*	*p*	η_p_^2^
Group	1	50.91	1.61	0.21	0.03
Presentation	**1**	**3102.82**	**5.69**	**0.02**	**0.002**
Noise level	1	**3107.99**	**1645.58**	** < 0.001**	**0.35**
Semantic content	2	2037.59	0.40	0.67	< 0.001
Group × Presentation	1	3099.98	0.01	0.93	< 0.001
Group × Noise level	1	3101.33	2.66	0.10	< 0.001
Presentation × Noise level	**1**	**3100.30**	**28.32**	** < 0.001**	**0.009**
Group × Semantic content	**2**	**3122.96**	**4.18**	**0.02**	**0.003**
Presentation × Semantic content	**2**	**3102.04**	**4.48**	**0.01**	**0.003**
Noise level × Semantic content	**2**	**3106.57**	**15.46**	** < 0.001**	**0.01**
Group × Presentation × Noise level	1	3101.24	0.45	0.50	< 0.001
Group × Presentation × Semantic content	2	3101.41	0.57	0.57	< 0.001
Group × Noise Level × Semantic content	2	3102.14	0.69	0.50	< 0.001
Presentation × Noise Level × Semantic content	2	3100.78	2.98	0.05	0.002
Group × Presentation × Noise level × Semantic content	2	3102.05	0.20	0.82	< 0.001

NumDF, numerator degree of freedom; DenDF, denominator degree of freedom; Presentation, mode of presentation. Significant effects and interactions are in bold

**Fig. 4:**
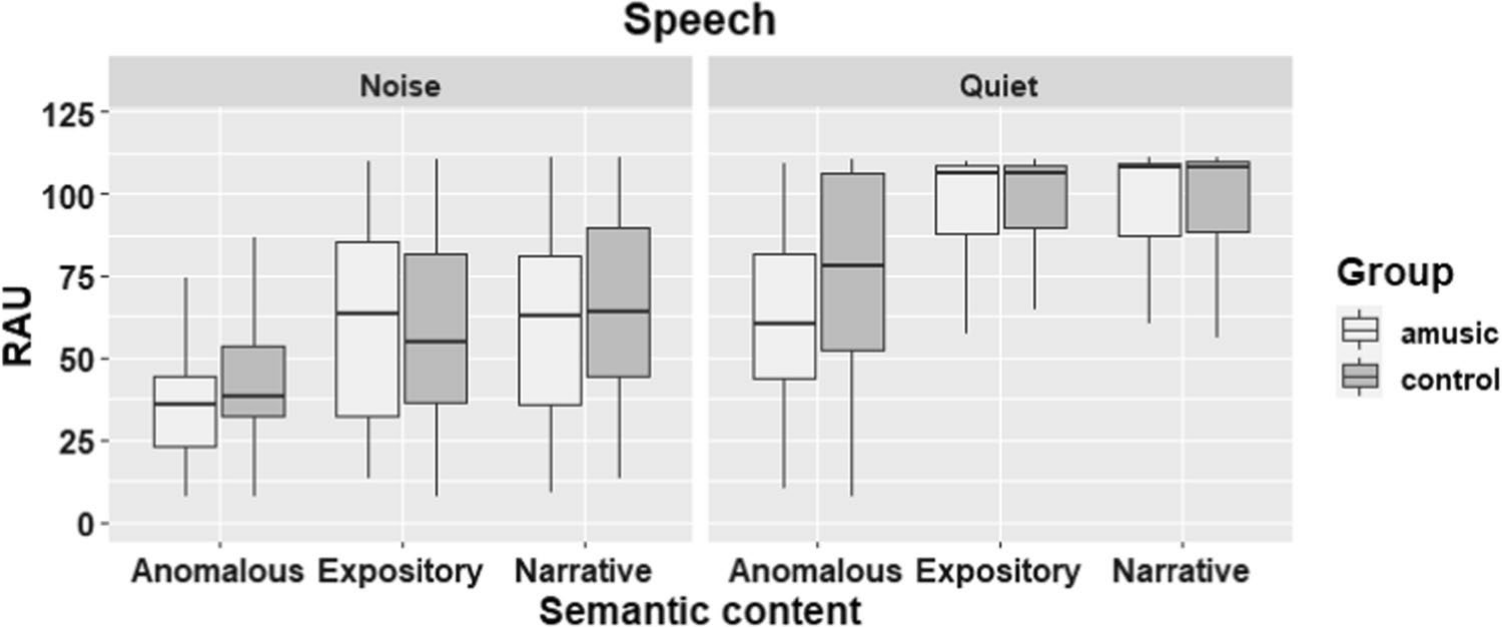
Performance of amusic participants and controls on all trials of the speech condition. The boxplots show the distribution of the data including the median and the quartiles

**Fig. 5:**
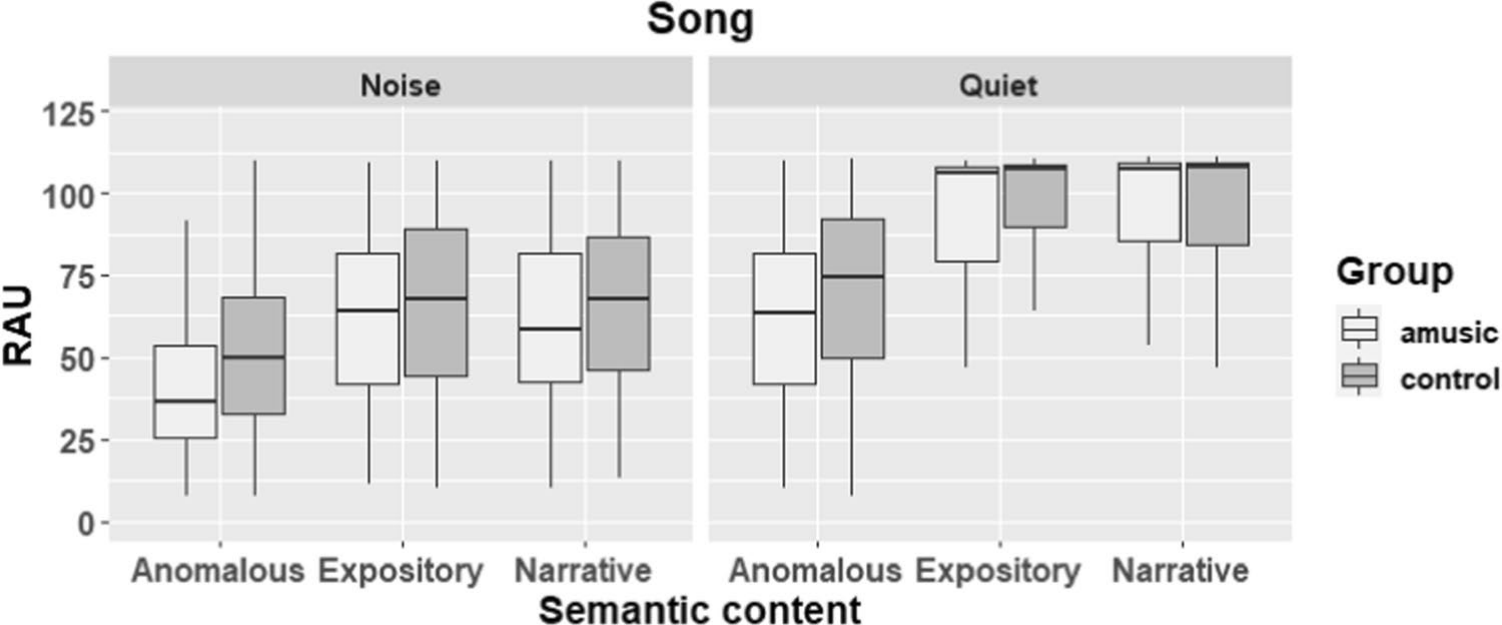
Performance of amusic participants and controls on all trials of the song condition. The boxplots display summary statistics including the median and the quartiles

The results of this group comparison are also visually displayed in Fig. 4 (speech condition) and Fig. 5 (song condition).

## Discussion

The current study provides an account of immediate repetition of spoken and sung utterances of varying semantic content with and without babble noise in musicians and non-musicians. A significant difference in performance between musicians and non-musicians was observed when amusia was factored out; however, identifying and excluding amusic participants led to no significant differences between the two groups. Overall, noise had an adverse effect on performance, with both musicians and non-musicians performing better in quiet. Sung targets were recalled more successfully than spoken ones in the presence of noise. Better performance was associated with sung expository relative to spoken expository utterances. Expository sentences were better recalled than anomalous ones but only in noise. Overall, the current results suggest that musicianship may not facilitate speech perception in noise, but undiagnosed deficits can introduce additional variability in speech and music processing.

A novel aspect of the study was that all participants were screened for amusia using the full MBEA battery (Peretz et al. 2003). This enabled a further distinction within the sample between those having an unimpaired perception of musical sounds and those having a music perception deficit (and perhaps comorbid speech processing difficulties; see, for example, Liu et al. 2015). The analysis of a pool of 100 participants showed a statistically significant main effect of group, with musicians recalling more words overall in comparison with non-musicians. However, inspection of the MBEA results revealed that 13 participants had amusia. Rerunning the model without these participants pointed to no significant group differences. This approach suggests that, unless amusia is ruled out, samples may contain atypical cases. Notably, this seems to occur at a higher rate than in the general population, estimated between 1.5 and 4% (Kalmus and Fry 1980; Peretz and Vuvan 2017), considering that the call for participation explicitly requires no (or very little) music training for half of the participants. Inadvertently including amusic participants may lead to less representative control groups across studies, with the musician/non-musician comparison being handicapped by unexpected variation in the control group. Hence, screening for amusia is arguably a crucial step to be taken in order to exclude such eventuality. It is worth noting, however, that comparing amusic participants to non-musician controls did not lead to a significant difference in performance. This finding diverges from previous work showing speech comprehension difficulties in amusia in the presence and absence of noise (Liu et al. 2015). Future studies are warranted to establish whether and on what stimulus complexity level amusic individuals may experience such difficulties, while also exploring possible subgroups in the amusia population.

Turning to music training and speech-in-noise perception, no musicianship advantage was observed in line with several previous studies (Boebinger et al. 2015; Hsieh et al. 2022; Madsen et al. 2017, 2019; Ruggles et al. 2014; Yeend et al. 2017). Inevitably, there is some variation in the design of previous studies that have (Meha-Bettison et al. 2018; Parbery-Clark et al. 2009a, b; Slater et al. 2015) or have not (Boebinger et al. 2015; Madsen et al. 2017, 2019; Ruggles et al. 2014; Yeend et al. 2017) found differences between musicians and non-musicians in this regard. It is also worth noting that musicians are a highly diverse group and the strict musician/non-musician dichotomy often fails to capture additional variation (Walsh et al. 2021). Speech-in-noise perception, in particular, has been associated with differences in performance depending on the type of music training; groups of percussionist and vocalists have been found to moderately outperform each other depending on whether the QuickSIN or the WIN (Words-In-Noise) test is used (Slater and Kraus 2016).

When the targets were sung—a novel aspect of this study—participants performed better. In their naturally occurring form, melodies typically involve markedly fewer notes per second relative to the syllables uttered per second in the speech domain (Kilgour et al. 2000; Patel 2014). Listeners may also pick up on melodic and rhythmic information found in singing but not in speech (Sloboda 1985). A more generous time window and additional cues may have afforded listeners in our study with a better opportunity for immediate recall. It is, however, of note that an advantage for repeating sung targets was observed only in the presence of noise, which suggests that participants benefitted from the above-mentioned acoustic parameters only in adverse auditory conditions. Recalling more sung words (relative to spoken ones) solely due to timing differences—rather than properties intrinsic to music—remains an open possibility. As we aimed for ecologically valid stimuli, we did not match the duration of the sung stimuli to that of the spoken ones. Further research is needed to narrow down the effects leading to this processing difference. A limitation of the study was that the fundamental frequency of spoken targets was not closely matched to that of sung ones. Hence, the difference in performance could be attributed to sung sentences standing out from the background more clearly.

When targets were presented with babble noise, expository sentences were associated with better performance relative to anomalous ones. This is in line with previous sentence repetition studies showing that children are more accurate in repeating semantically plausible sentences (Polišenská et al. 2015, 2021). Our results also concur well with previous work showing a positive effect of typical semantic expectancies in adverse listening conditions in participants without music training (Golestani et al. 2010; Obleser and Kotz 2010). We did not observe better recall of narrative versus expository sentences in contrast to what is typically seen in recall of written language (Kintsch and Young 1984; Mar et al. 2021; Zabrucky and Moore 1999). However, not all studies have observed the same difference between these semantic categories (Roller and Schreiner 1985), and it is also not known to what extent participants’ prior knowledge may contribute to these results (Cunningham and Gall 1990; Wolfe 2005; Wolfe and Mienko 2007). Recall ability of those with more extensive knowledge can, in fact, be more enhanced in the case of expository content (Wolfe and Mienko 2007). It is not clear why semantic plausibility did not affect performance in quiet. Further work is needed to establish the threshold for repetition performance differences in relation to sentence plausibility.

The absence of a significant difference between musicians and non-musicians in disentangling musical targets from noise is, at least on first consideration, surprising. Previous evidence suggests that, when presented with harmonic complexes, musicians perform better at auditory stream segregation (Zendel and Alain 2009). Further, when the acoustic signal comprises only two speakers, one being the target and the other the distractor, musicians have been also found to outperform non-musicians (Başkent and Gaudrain 2016), perhaps owing to musicians’ enhanced pitch discrimination ability (Micheyl et al. 2006). However, these results could be partly attributed to musicians’ ability to better perceive interleaved melodies (Marozeau et al. 2010), which is not what was tested in the present study. An area of further exploration could be to test musicians and untrained controls in their ability to segregate a target sung sentence from a distractor sung sentence. Another remaining question is whether there would be differences in performance if our participants reproduced sentences in the target modality (that is, singing for sung sentences and speech for spoken ones). However, a challenge in such design would be to disentangle singing ability (arguably better in musicians) and confidence stemming thereof from recall performance.

Music training (in years) correlated significantly with the MBEA but not with the other cognitive measures obtained in this study. Previous work has shown links between music training and various non-musical cognitive abilities, such as visuospatial processing (Brochard et al. 2004; Gagnon and Nicoladis 2021; Hetland 2000; Sluming et al. 2007), executive functioning (Bialystok and DePape 2009; Pallesen et al. 2010), memory (Talamini et al. 2017) and mathematics, to a degree (Vaughn 2000). Nonetheless, other studies have cast doubts on the effect of musicianship on non-musical cognitive abilities (McKay 2021; Mehr et al. 2013; Rickard et al. 2012; Rodrigues et al. 2014; Sala and Gobet 2020). The use of different tasks across studies may be a viable explanation for such inconsistencies.

Overall, this work has not lent support to theories entertaining musicianship as a facilitative factor in speech perception in noise or in quiet, when amusia is accounted for. The current study points to various interactions—perhaps indicative of the intricacies of sentence processing—that would not be detected using a simpler design. At the same time, the study suggests that settling the question of musicianship’s contribution may be hindered by recruiting unrepresentative samples, thus making a case for wider use of amusia screening in psycholinguistics/music psychology studies. Determining the contribution of relevant variables—acoustic, semantic, disorder-related or otherwise—seems worthy of further investigation.
